# A Bayesian machine learning approach to Nature vs. Nurture: Predicting a cognitive marker of Alzheimer’s risk in a deeply‐phenotyped cohort of cognitively unimpaired older African Americans

**DOI:** 10.1002/alz.091357

**Published:** 2025-01-09

**Authors:** Bernadette A. Fausto, Miray Budak, Mustafa Sheikh, José Mojica Perez, Shengguo Li, Liangyuan Hu, Mark A. Gluck

**Affiliations:** ^1^ Rutgers University–Newark, Newark, NJ USA; ^2^ Rutgers University, New Brunswick, NJ USA; ^3^ Rutgers School of Public Health, Piscataway, NJ USA

## Abstract

**Background:**

Late‐onset Alzheimer’s disease (AD) arises from interactions between genetic (nature) and environmental/behavioral (nurture) factors, but their relative contributions are not well defined. With the development of sensitive cognitive tasks for early detection and the growing applications of machine learning to AD research, there are unprecedented opportunities to uncover the most salient genetic and environmental/behavioral factors for preclinical AD risk. Our previous studies show that generalization performance––the ability to apply prior learning to new contexts––is a cognitive risk marker for preclinical AD. Using a novel technique combining Bayesian machine learning and bootstrap imputation, the present study identified among various candidate genetic and environmental/behavioral factors which most strongly predicted generalization performance in a deeply‐phenotyped cohort of cognitively unimpaired older African Americans.

**Method:**

472 older African American participants from the Pathways to Healthy Aging in African Americans cohort study (Mage=68.34 years, SD=6.99; Meducation=13.93 years, SD=2.36; MMMSE=27.65, SD=1.95) responded to demographic, health, and lifestyle questionnaires; provided a saliva sample for the following AD risk genotyping: ABCA7 rs115550680, APOE‐ε4 status, ABCA7 rs3764650; and completed a cognitive battery including a generalization task (Concurrent Discrimination and Transfer Task). We employed Bayesian Additive Regression Trees (a.k.a., BART) with a bootstrap imputation‐based variable selection procedure to extract the top three variables in terms of relative contribution to generalization performance.

**Result:**

Among 25 candidate variables, the top three ranked variables related to generalization included ABCA7 rs115550680, est.=3.29 [95% CI 0.42, 6.19]; APOE‐ε4 status, est.=2.41 [95% CI 0.37, 4.46]; and their performance on the training phase of the generalization task, est.=0.92, [95% CI 0.86, 0.99], above and beyond other demographic, health, lifestyle, and cognitive variables.

**Conclusion:**

Using novel machine learning techniques and a sensitive cognitive risk marker, we identified that generalization performance is most strongly driven by genetic vulnerability to AD among older African Americans. These findings suggest a greater contribution of genetic factors (nature) compared to environmental/behavioral factors (nurture) to late‐onset AD risk within this population.

